# Modulated Radio Frequency Stealth Waveforms for Ultra-Wideband Radio Fuzes

**DOI:** 10.3390/e26070605

**Published:** 2024-07-17

**Authors:** Kaiwei Wu, Bing Yang, Shijun Hao, Yanbin Liang, Zhonghua Huang

**Affiliations:** School of Mechatronical Engineering, Beijing Institute of Technology, Beijing 100081, China; wukaiwei@bit.edu.cn (K.W.); 3120215164@bit.edu.cn (B.Y.); 3120215165@bit.edu.cn (S.H.); liangyanbin@bit.edu.cn (Y.L.)

**Keywords:** radio frequency (RF) stealth, chaotic, ultra-wideband, fuze

## Abstract

The increasingly complex electromagnetic environment of modern warfare and the proliferation of intelligent jamming threaten to reduce the survival rate of radio fuzes on the battlefield. Radio frequency (RF) stealth technology can fundamentally improve the anti-interception and reconnaissance capabilities of radio fuzes, thereby lessening the probability of them being intercepted, recognized, and jammed by the enemy. In this paper, an RF stealth waveform based on chaotic pulse-position modulation is proposed for ultra-wideband (UWB) radio fuzes. Adding a perturbation signal based on the Tent map ensures that the chaotic sequences have sufficiently long periods despite hardware byte limitations. Measuring the approximate entropy and sequence period shows that the Tent map with the addition of perturbation signals can maintain good randomness under byte constraints, closely approximating the Tent map with ideal precision. Simulations verify that the proposed chaotic mapping used to modulate the pulse position of an ultra-wideband radio fuze signal results in superior detection, anti-interception, and anti-jamming performance.

## 1. Introduction

### 1.1. Radio Fuzes

A radio fuze is a device that utilizes environmental or target information to control the detonation of ammunition according to a predetermined strategy. The effectiveness of the radio fuze determines the effectiveness of the munition to which it is attached. To protect their positions, enemies typically deploy jammers around the perimeter, to intercept fuze signals and transmit interference signals, thereby disrupting radio fuzes. The rapid development of radio frequency hardware circuits and signal processing technology has significantly improved jammer capabilities. Various interference waveforms have been designed that seriously threaten the battlefield survivability of radio fuzes [1,2,3,4]. Suppressive interference waveforms can drown the echo signal in dense electromagnetic waves, preventing the fuze from obtaining and extracting distance information in time, which results in misfires. Targeted interference waveforms can serve as false target information. When the fuze receives an interference signal, it may misjudge it as target information and detonate prematurely. Both misfires and premature detonations result in the ammunition failing to achieve its maximum effectiveness. Therefore, improving the ability of radio fuzes to survive on the battlefield is an important priority in military research.

**Figure 1:**
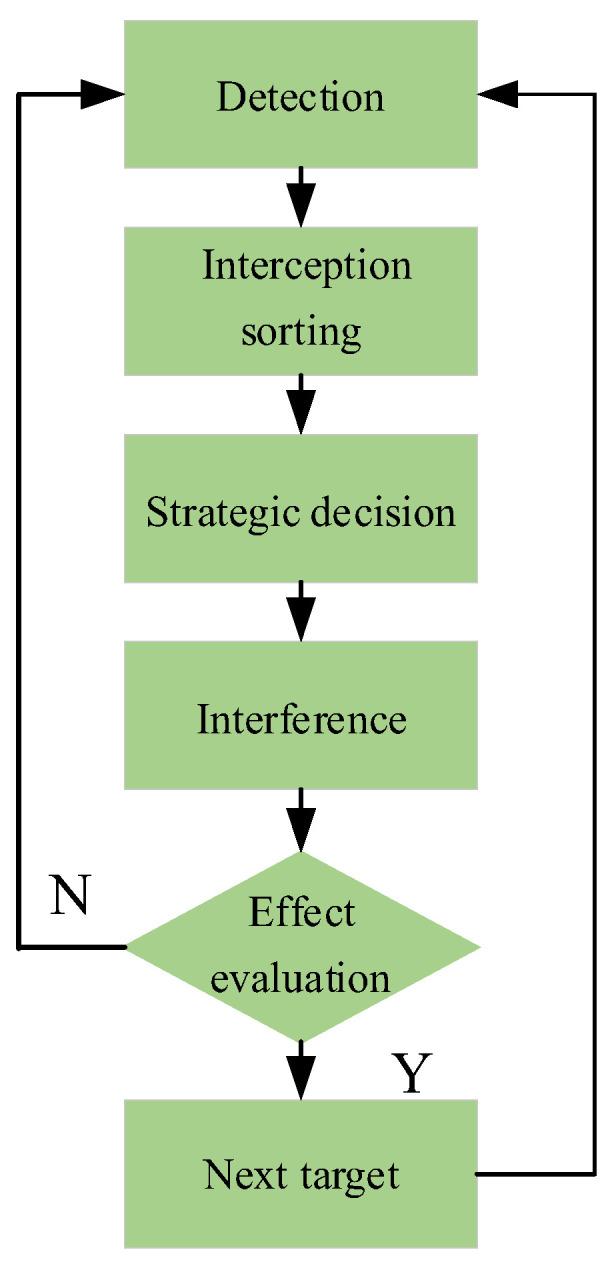
Interference process.

Figure 1 illustrates the process of radio fuze jammers interfering with radio fuzes. To implement effective jamming, the first step is to detect the radiated signals of the radio fuzes, followed by sorting the detected signals, i.e., detecting the operating frequency of the signals and analyzing their characteristics. A specific jamming method and waveform are then selected based on the signal characteristics. After the interference is executed, its effect is evaluated; if the interference fails, the process is repeated, and if the interference succeeds, the next target to be jammed is identified [5]. The jammer’s threat to radio fuzes is based on its ability to detect the radio fuze signal. The application of RF stealth technology in radio fuze design can prevent such detection, thereby stopping jammers from interfering with the fuze at its source.

RF stealth was initially proposed by David Lynch [6], who discussed various methods to minimize the RF signal characteristics of radar, communication, and weapon systems aboard aircraft. This approach aims to make it challenging for enemy electronic reconnaissance systems to detect and promptly identify the radiation source. For radio fuzes, the objective of RF stealth is to decrease the likelihood of interception, detection, identification, and subsequent jamming of radio fuze signals by enemy jammers. Therefore, in designing RF stealth radio fuzes, two primary approaches are considered. One approach involves reducing the radiation power of the radio fuze to hinder signal detection by jammers. The other approach focuses on increasing the randomness of the radio fuze’s signals. This strategy ensures that even if the jamming device detects the signals, it cannot accurately recognize them or proceed with further processing [7].

Currently, the most commonly employed RF stealth techniques include radiated power control and stealth waveform design [8]. Several studies [9,10,11,12] have concentrated on power control, which involves optimizing the emitted signal strength to evade interception and recognition by jammers, while still being robust enough to detect targets in complex electromagnetic environments. Similarly, waveform design aims to balance the signal’s resilience against interception with its localization accuracy [13], resolution capability [14], and ability to mitigate delay-Doppler blurring of potential targets [15]. One method to achieve this balance is interference-pulse anti-sorting [16,17,18], which introduces interference pulses to the signal, complicating the enemy receiver’s ability to identify it from the pulse-repetition-interval information it contains. Another approach involves randomly or chaotically modulating certain signal parameters [19,20,21,22,23], making it challenging for jammers to recognize the signal.

Current anti-jamming methods for radio fuzes mainly use multi-dimensional feature recognition and other techniques to enhance fuze resilience against jamming [24,25]. However, digital radio frequency memory (DRFM) jammers differ significantly from traditional targeting [26] and sweeping jammers [27,28]. DRFM jammers intercept and store fuze transmission signals, then retransmit them after delay with amplification [29]. The interference waveform produced by DRFM jammers is nearly indistinguishable from the radio fuze echo signal, even with multi-dimensional feature recognition techniques. Thus, designing waveforms with RF stealth capabilities is crucial to prevent jammer interception of fuze signals. UWB signals exhibit minimal interception vulnerability in the frequency domain [30], and appear as periodic, extremely narrow pulse signals in the time domain. Modulation techniques using pseudo-random codes like M-sequences are constrained by their inherent periodicity. However, chaotic codes characterized by sensitivity to initial values, non-periodicity, and randomness, represent an emerging pseudo-code. Utilizing chaotic codes for UWB radio fuze pulse position modulation increases waveform parameter complexity and enhances anti-interception capabilities.

### 1.2. Ultra-Wideband Radio Fuzes

This study focuses specifically on UWB radio fuzes. These fuzes detect ground targets by transmitting and receiving narrow pulse signals. They possess strong anti-jamming capabilities, superior distance-truncation characteristics, and robust anti-interception capabilities [31]. Their simple structure, wide spectral range, and low power-spectral density make interception by the enemy challenging [32].

**Figure 2:**
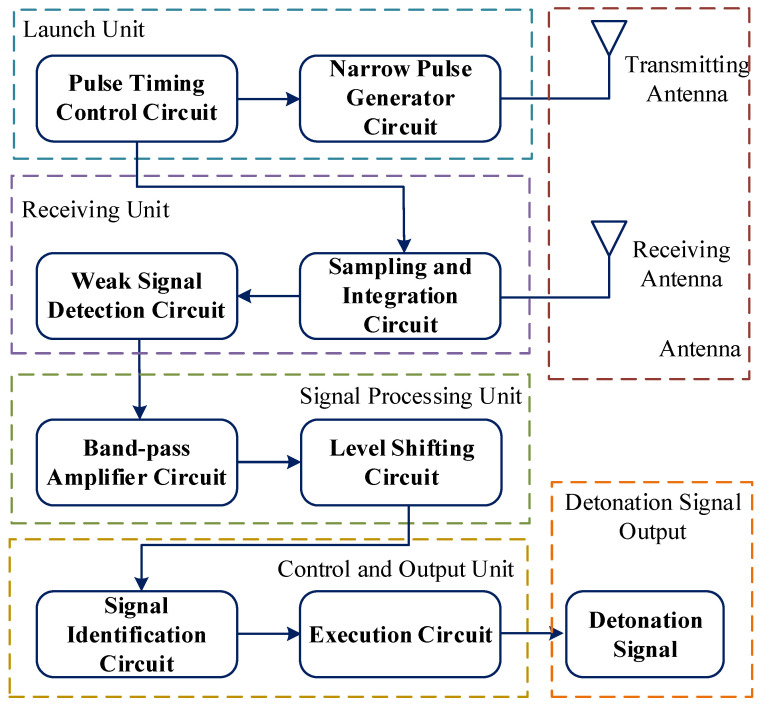
Operation of UWB radio fuze.

Figure 2 depicts the principal block diagram of the radio fuze, consisting mainly of the transmitting unit, receiving unit, signal processing unit, control and output unit, and antenna. The transmitting unit includes a pulse-timing control circuit, which triggers the narrow pulse generator circuit to produce nanosecond-wide pulses transmitted via the antenna. Simultaneously, it serves as a distance control gate, triggering the sampling pulse generator circuit to generate sampling pulses after a predetermined delay. The receiving unit comprises a sampling pulse circuit and a weak signal detection circuit. When the distance between the munition and the ground target matches the fuze’s preset detonation height, the sampling pulse circuit controls the weak signal detection circuit to sample the received echo signal from the antenna. The output signal from the receiving circuit undergoes processing in the signal processing unit, with the processed signal forwarded to the control and output unit. The control and output unit initiates the fuze by outputting the start signal.

In this study, the transmitter signal *w*(*t*) is defined as the second derivative of the Gaussian function as follows:

(1) $$w(t)=\left(1-\frac{2\pi t^{2}}{\Delta T^{2}}\right)\exp\left[-\pi (\frac{t}{\Delta T}{)}^{2}\right],$$

where Δ*T* is the pulse width parameter.

With the inclusion of pulse-position modulation, the mathematical expression of the transmitted signal becomes the following:

(2) $$s(t)=\sum_{i=-\infty }^{+\infty }w(t-iT-c_{i}T_{0}),$$

where T is the pulse period, $c_{i}$ is a random sequence, $T_{0}$ is the modulation range, and ${c_{i}T}_{0}$ is a uniformly distributed random variable in ${[0,T}_{0}]$.

### 1.3. Chaos Theory

Chaos is a phenomenon that introduces irregular and stochastic behavior into deterministic systems. Its initial value sensitivity and intrinsic randomness make it very suitable for designing random signals. Even with deterministic chaotic mapping, distinct initial values lead to entirely different chaotic trajectories over time. Unlike purely random noisy signals, deterministic chaotic sequences can be reconstructed from known parameters and initial values and synchronized for simplified signal processing. Unlike pseudo-random signals, they are non-periodic, generate numerous distinct sequences, and can extend infinitely in length. Utilizing chaotic sequences to modulate pulse positions in UWB fuzes enhances the signal’s resistance to interception and complicates decipherment by adversaries [33].

Chaotic signals can be generated by continuous-time dynamic analog systems like Chua’s circuit, but these analog systems tend to be bulky and susceptible to environmental factors. However, radio fuzes must adhere to strict volume constraints of projectiles, necessitating compact design and high reliability. Thus, digital devices are preferred for timing control circuit design. However, byte limitations in digital devices lead to short-period phenomena when generating chaotic signals. Consequently, the resulting signal becomes periodic and only chaotic. For practical applications, chaotic sequences should ideally have extended periods to maximize decipherment difficulty and waveform interception resistance.

Owing to operational and memory constraints in practical engineering, one-dimensional chaotic mappings are typically favored. Equation (3) is the classical Tent map.

(3) $$x_{n+1}=\left\{\begin{array}{cc} \frac{x_{n}}{\alpha } & x_{n}\in \left[0,\alpha \right)\\ \frac{1-x_{n}}{1-\alpha } & x_{n}\in \left[\alpha ,1\right) \end{array}\right.\begin{array}{cc} & \alpha \in \left(0,1\right) \end{array},$$

and Equation (4) is the classical Logistic map.

(4) $$x_{n+1}=\mu x_{n}\left(1-x_{n}\right)\begin{array}{cc} & \mu \in \left[0,4\right] \end{array}.$$

**Figure 3:**
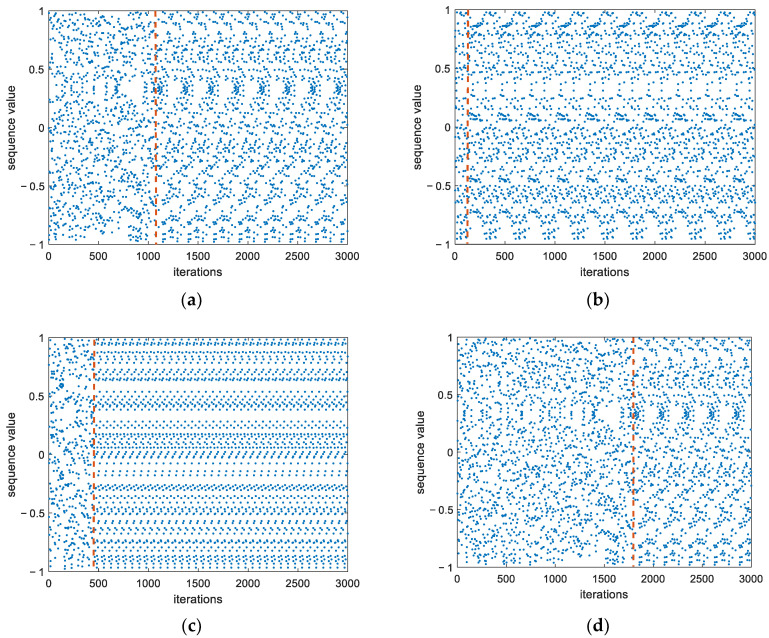
Tent mapping with different initial values (*p* = 6) showing the emergence of regularity after a small number of iterations. (**a**) initial value $x_{0}=0.35$; (**b**) initial value $x_{0}=0.33$; (**c**) initial value $x_{0}=0.68$; (**d**) initial value $x_{0}=0.85$.

Under byte-constrained conditions, a chaotic sequence degenerates into a periodic one. For instance, as depicted in Figure 3 for the Tent map, retaining six bits after the decimal point and using different initial values results in the Tent map degenerating into a periodic sequence during iteration, where the red dashed line indicates the starting position of degeneration into a periodic sequence. This periodicity makes it easier for adversaries to decipher because of its regularity in signal modulation.

## 2. Method

### 2.1. Perturbation Parameter

In this paper, we introduce a perturbation parameter r connecting the logistic map to the Tent map as follows:

(5) $$x_{n+1}=\left\{\begin{array}{cc} \frac{x_{n}}{a}+r*y_{n} & x_{n}\in \left[0,a\right)\\ \frac{1-x_{n}}{1-a}+r*y_{n} & x_{n}\in \left[a,1\right) \end{array}\right.,$$

(6) $$y_{n+1}=\mu y_{n}\left(1-y_{n}\right)\begin{array}{cc} & \mu \in \left[0,4\right] \end{array},$$

(7) $$r=\left\{\begin{array}{cc} 0.0001 & \mathrm{mod}(n,P)=0,P=100\\ 0 & \end{array}\right..$$

Chaotic sequences are highly sensitive to initial values, where even slight differences can lead to entirely divergent trajectories over iterations. The addition of the periodicity perturbation parameter can disrupt the closed state set of the original chaotic system, thereby eliminating short-cycle phenomena. To minimize interference with the original chaotic sequence, the signal-to-noise ratio between the original signal and the perturbation signal should significantly exceed unity [34]. Therefore, we chose a perturbation parameter as small as possible within the limits of accuracy to prevent excessive perturbation and ensure the chaotic sequence remains within its intended range. For this study, we chose 1 × 10^−4^ as the value of r.

**Figure 4:**
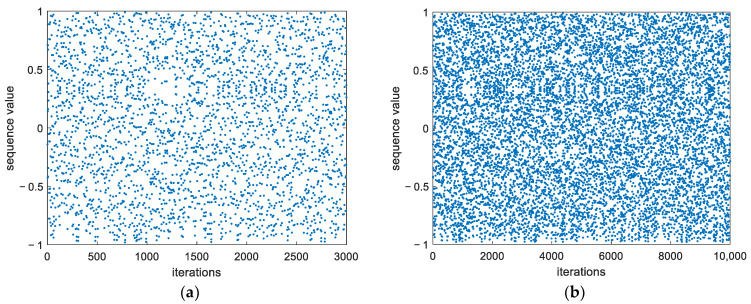
Tent mapping with perturbation parameter added (*p* = 6). Note the absence of regularity even after 10,000 iterations: (**a**) Tent mapping after 3000 iterations; (**b**) Tent mapping after 10,000 iterations.

The approximation to chaos used in this study produces the map in Figure 4 when six bits are retained after the decimal point. Figure 4a illustrates the chaotic scatter plot after 3000 iterations, showing no observable periodic phenomena. Even with an increased iteration count of 10,000, as shown in Figure 4b, it still maintains good chaotic characteristics.

### 2.2. Approximate Entropy as a Measure of Complexity

In this study, the approximate entropy is used to measure the complexity of chaotic sequences [35]. This metric reflects the likelihood of generating a new sequence within an existing one: a higher approximate entropy value indicates greater complexity. When dealing with a known sequence $x\left(i\right)$, the algorithm for calculating the approximate entropy is as follows:

(1) Convert the sequence $x\left(i\right)$ into an m-dimensional vector $\alpha \left(i\right)$:

(8) $$\alpha \left(i\right)=\left[x\left(i\right),x\left(i+1\right),\cdots x\left(i+m-1\right)\right].$$

(2) Define $\;d\left[\alpha \left(i\right),\alpha \left(j\right)\right]$ as the maximum difference between the corresponding elements of $\alpha \left(i\right)$ and $\alpha \left(j\right)$:

(9) $$d\left[\alpha \left(i\right),\alpha \left(j\right)\right]=\underset{k=1,2,\cdots ,m}{max}\left|x\left(i+k-1\right)-x\left(j+k-1\right)\right|.$$

(3) Let *s* denote the similarity tolerance. Then, for every value of i (including i=j) 1≤i≤N−m+1, count the number of vectors that satisfy $d\left[\alpha \left(i\right),\alpha \left(j\right)\right]\leq s$. Let $C_{i}^{m}\left(s\right)$ denote the ratio of this number to the total number of *α*-vectors:

(10) $$C_{i}^{m}\left(s\right)=\frac{1}{N-m-1}num\left\{d\left[\alpha \left(i\right),\alpha \left(j\right)\right]\leq s\right\}.$$

(4) Calculate the Napierian logarithm of $C_{i}^{m}\left(s\right)$ and its mean value $\Phi^{m}\left(s\right)$:

(11) $$\Phi^{m}\left(s\right)=\frac{1}{N-m-1}\sum_{i-1}^{N-m-1}\ln(C_{i}^{m}(s)).$$

(5) Finally, define the approximate entropy of the sequence as follows:

(12) $$E(m,s,N)=\Phi^{m}(s)-\Phi^{m+1}(s),m\geq 2.$$

## 3. Results and Discussion

### 3.1. Comparison of Complexity

**Figure 5:**
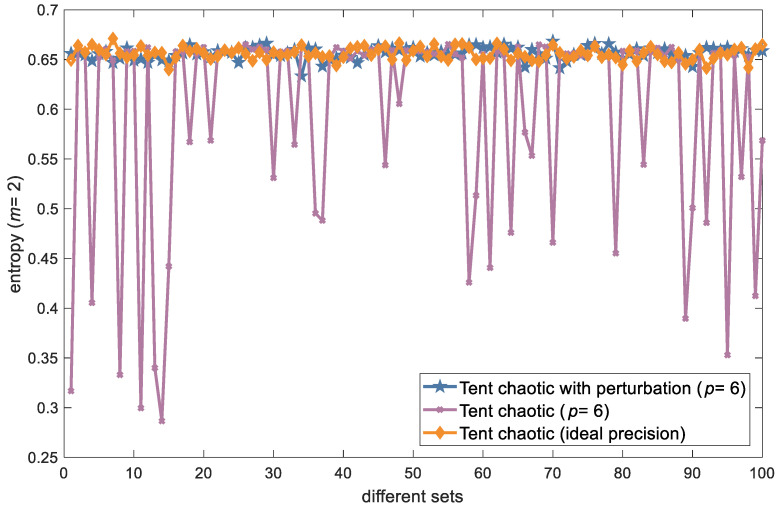
Comparison of the approximate entropies of three Tent-chaotic sequences.

We calculated the approximate entropy in two dimensions for the Tent chaotic output sequence at ideal precision and compared it to those at finite precision (*p* = 6), both with and without the added perturbation parameter. Figure 5 illustrates the approximate entropy of these output sequences of the three chaotic systems, evaluated across 100 randomly selected sets of initial values. As depicted, the approximate entropy of the Tent-chaotic sequence remained nearly unchanged with the addition of the perturbation parameter under finite accuracy, closely resembling the entropy of the ideal accuracy scenario without perturbation. By contrast, without the perturbation parameter, the sequence’s approximate entropy exhibited instability and significantly lower complexity in certain initial value cases.

### 3.2. Comparison of Period Length

**Figure 6:**
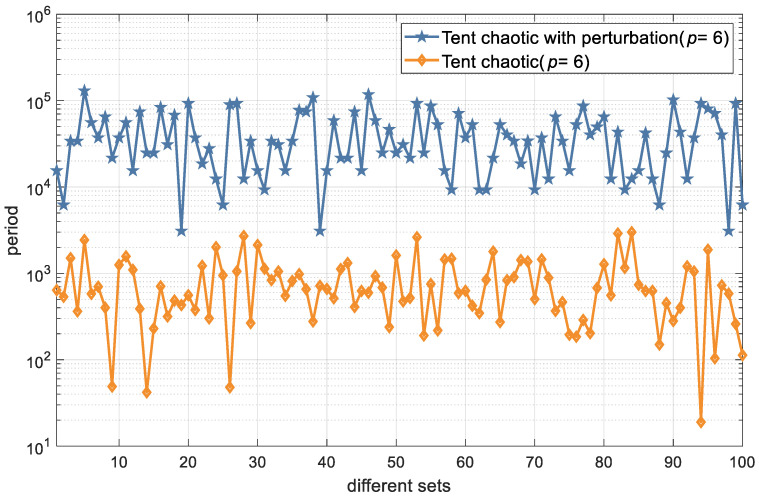
Period lengths of Tent-chaotic sequences with and without added perturbation parameter.

For precision *p* = 6, we calculated and compared the period length of 100 groups of chaotic maps with different initial values (Figure 6). It can be seen that the periods of the Tent chaotic sequences with the perturbation parameter are much larger than those of sequences without it.

### 3.3. Fuze Performance Analysis

#### 3.3.1. Detection Performance

The ambiguity function (AF), initially proposed by P.M. Woodward to describe radar-modulation waveform characteristics, serves to depict not only the resolution of radar signals and the ambiguity levels but also measurement accuracy and clutter suppression [36]. Radio fuzes operate similarly to radar systems, allowing the AF to describe their detection performance. The AF evaluates a signal’s ability to distinguish between two targets at varying distances and velocities. In this study, we utilized the mean square error criterion to derive the ambiguity function for UWB signals.

For an ideal point target 1 with a time delay x and Doppler shift y, by ignoring antenna effects on the waveform of the transmitted signal, the echo signal is expressed as follows:

(13) $${u_{r}}_{1}\left(t\right)=\sum_{i=0}^{N-1}w\left[t-x-i\left(T-y\right)\right].$$

For another ideal point target 2, with additional time delay τ and Doppler shift $T_{d}$ relative to reference target 1, the expression for the echo signal becomes the following:

(14) $$u_{r_{2}}\left(t\right)=\sum_{i=0}^{N-1}w\left[t-\left(x+\tau \right)-i\left(T-y-T_{d}\right)\right].$$

The mean square deviation of the two echoes is as follows:

(15) $$\begin{array}{cc} \varepsilon^{2} & =\int_{-\infty }^{\infty }\;{\left|\sum_{i=0}^{N-1}\;w[t-x-i(T-y)]-\sum_{i=0}^{N-1}\;w[t-(x+\tau )-i(T-y-T_{d})]\right|}^{2}dt\\ & =\int_{-\infty }^{\infty }\;{\left|\sum_{i=0}^{N-1}\;w[t-x-i(T-y)]\right|}^{2}dt+\int_{-\infty }^{\infty }\;{\left|\sum_{i=0}^{N-1}\;w[t-(x+\tau )-i(T-y-T_{d})]\right|}^{2}dt\\ & -2\int_{-\infty }^{\infty }\;\sum_{i=0}^{N-1}\;w\left[t-x-i\left(T-y\right)\right]\sum_{i=0}^{N-1}\;w\left[t-\left(x+\tau \right)-i\left(T-y-T_{d}\right)\right]dt. \end{array}$$

The echo energies for ideal targets 1 and 2 are denoted as E1 and E2, respectively:

(16) $${E1=\int_{-\infty }^{\infty }\left|\sum_{i=0}^{N-1}w[t-x-i(T-y)]\right|}^{2}dt,$$

(17) $${E2=\int_{-\infty }^{\infty }\left|\sum_{j=0}^{N-1}w[t-(x+\tau )-j(T-y-T_{d})]\right|}^{2}dt.$$

By ignoring propagation losses, we assume $E_{1}=E_{2}=E^{\prime }$. If $t-(x+\tau )=t^{\prime }$, Equation (15) simplifies to the following:

(18) $$\varepsilon^{2}=2E-2\int_{-\infty }^{\infty }\sum_{i=0}^{N-1}w\left[t+\tau -i\left(T-y\right)\right]\sum_{i=0}^{N-1}w\left[t-i\left(T-y-T_{d}\right)\right]dt.$$

By setting y=0 (i.e., there is no Doppler shift for target 1), the AF of an unmodulated UWB pulse train is defined as follows:

(19) $$\chi \left(\tau ,T_{d}\right)=\int_{-\infty }^{\infty }\sum_{i=0}^{N-1}w\left[t+\tau -iT\right]\sum_{i=0}^{N-1}w\left[t-i\left(T-T_{d}\right)\right]dt.$$

Equation (19) shows that using the AF of an unmodulated UWB pulse train transforms the problem of discriminating targets based on different distances and velocities into that of discriminating the time delay and Doppler shift of the echo signals.

Similarly, for chaotic pulse position modulation, for the ideal point target 1, the echo signal with time delay x is given by the following equation:

(20) $${u_{r}}_{1}\left(t\right)=\sum_{i=0}^{N-1}w[t-x-iT-C_{i}T_{0}+\sum_{j=1}^{i}\frac{2T_{j}v_{1}}{c})]\;\;=\sum_{i=0}^{N-1}w[t-x-iT-C_{i}T_{0}+\frac{2(iT+C_{i}T_{0}-X_{0})v_{1}}{c})].$$

For an ideal point target 2, with time delay τ and approach speed $v_{2}$ relative to reference target 1, the echo signal is given by the following:

(21) $$u_{r_{2}}\left(t\right)=\sum_{i=0}^{N-1}w\left[t-\left(x+\tau \right)-iT-C_{i}T_{0}+\frac{2\left(iT+C_{i}T_{0}-X_{0}\right)\left(v_{1}+v_{2}\right)}{c}\right].$$

The mean square deviation of the two echo signals is as follows:

(22) $$\begin{array}{cc} \varepsilon^{2} & =\int_{-\infty }^{\infty }\;{\left|\sum_{i=0}^{N-1}\;w[t-x-iT-C_{i}T_{0}+\frac{2\left(iT+C_{i}T_{0}-X_{0}\right)v_{1}}{c}]-\sum_{i=0}^{N-1}\;w[t-(x+\tau )-iT-C_{i}T_{0}+\frac{2\left(iT+C_{i}T_{0}-X_{0}\right){(v}_{1}+v_{2})}{c}]\right|}^{2}dt\\ & =\int_{-\infty }^{\infty }\;{\left|\sum_{i=0}^{N-1}\;w[t-x-iT-C_{i}T_{0}+\frac{2\left(iT+C_{i}T_{0}-X_{0}\right)v_{1}}{c}]\right|}^{2}dt+\int_{-\infty }^{\infty }\;{\left|\sum_{i=0}^{N-1}\;w[t-(x+\tau )-iT-C_{i}T_{0}+\frac{2\left(iT+C_{i}T_{0}-X_{0}\right){(v}_{1}+v_{2})}{c}]\right|}^{2}dt\\ & -2\int_{-\infty }^{\infty }\;\sum_{i=0}^{N-1}\;w\left[t-x-iT-C_{i}T_{0}+\frac{2\left(iT+C_{i}T_{0}-X_{0}\right)v_{1}}{c}\right]\sum_{i=0}^{N-1}\;w\left[t-(x+\tau )-iT-C_{i}T_{0}+\frac{2\left(iT+C_{i}T_{0}-X_{0}\right){(v}_{1}+v_{2})}{c}\right]dt. \end{array}$$

Expressing this in terms of variable *t’*, where $t-\left(x+\tau \right)=t^{\prime }$:

(23) $$\varepsilon^{2}=2E-2\int_{-\infty }^{\infty }\left|\sum_{i=0}^{N-1}w[t+\tau -iT-C_{i}T_{0}+\frac{2(iT+C_{i}T_{0}-X_{0})v_{1}}{c})]\sum_{i=0}^{N-1}w[t-iT-C_{i}T_{0}\;+\frac{2(iT+C_{i}T_{0}-X_{0})(v_{1}+v_{2})}{c}]\right|dt.$$

To simplify the discussion, let $v_{1}=0$ and $v_{2}=v$. The AF of a chaotic pulse-position-modulated UWB train can then be expressed as follows:

(24) $$\chi \left(\tau ,v\right)=\int_{-\infty }^{\infty }\sum_{i=0}^{N-1}w\left[t+\tau -iT-C_{i}T_{0}\right]\sum_{i=0}^{N-1}w\left[t-iT-C_{i}T_{0}\;+\frac{2\left(iT+C_{i}T_{0}-X_{0}\right)v}{c}\right]dt.$$

From the above analysis, it can be seen that, for the chaotic pulse-position-modulated UWB pulse train, the Doppler time shift varies among pulse-repetition cycles. Thus, the pulse train’s AF is not a function of delay and Doppler time shift but of delay and velocity.

**Figure 7:**
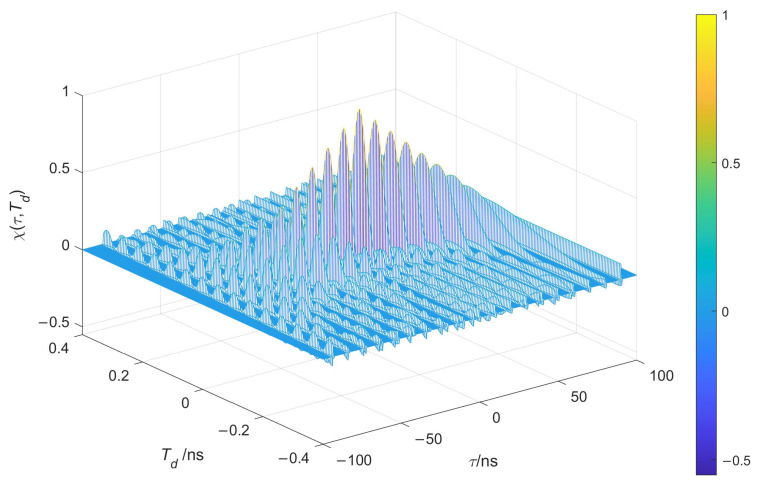
Ambiguity function of unmodulated UWB pulse train.

**Figure 8:**
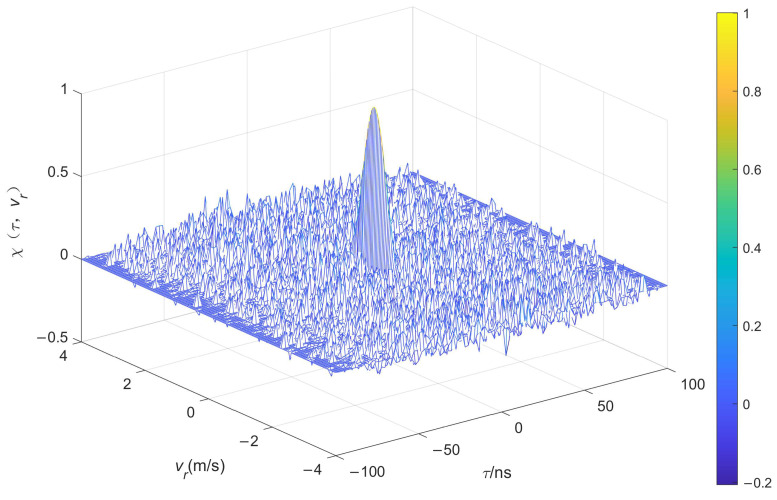
Ambiguity function of chaotic pulse-position-modulated UWB pulse train.

Based on the above theoretical derivation, the normalized AF diagrams of the unmodulated (Figure 7) and chaotic pulse-position-modulated (Figure 8) UWB train can be obtained, in which different colors are used to indicate the magnitude of the AF values.

As can be seen in Figure 7 and Figure 8, the AF of the chaotic pulse-position-modulated UWB pulse train approximates a thumbtack function, meaning that it has higher range accuracy and greater resistance to clutter interference than that of the unmodulated UWB pulse train.

#### 3.3.2. Anti-Interception Performance

The power spectrum S(f) of the signal s(t) of a UWB system consists of two parts, the discrete spectrum $S^{d}(f)$ and the continuous spectrum $S^{c}(f)$:

(25) $$S^{d}\left(f\right)=\frac{1}{T^{2}}{\sum_{l=-\infty }^{+\infty }{\left|W\left(\frac{l}{T}\right)\right|}^{2}\left|E\{\exp(j2\pi \frac{l}{T}{C_{i}T}_{0})\}\right|}^{2}\delta \left(f-\frac{l}{T}\right),$$

(26) $$S^{c}(f)=\frac{1}{T}{\left|W\left(f\right)\right|}^{2}\sum_{l=-\infty }^{+\infty }E\{[\beta_{n}(f)-E\{\beta_{n}(f)\}][\beta_{n+1}(f)-\;E\{\beta_{n+1}(f)\}{]}^{*}\}\exp(-2j\pi flT),$$

where $\beta_{n}(f)=\exp(j2\pi fC_{i}T_{0})$. Assuming $x_{n}$ is a chaotic sequence with $0<x_{n}<1,$ the probability density function of $c_{n}T_{0}$ is uniform: $\rho (C_{i}T_{0})=\frac{1}{T_{0}}$.

Consequently,

(27) $$E\{\exp(j2\pi fC_{i}T_{0})\}=\int_{0}^{T_{0}}\frac{1}{T_{0}}\exp(j2\pi fx)dx=\frac{\sin(\pi fT_{0})}{\pi fT_{0}}\exp(j\pi fT_{0}).$$

The continuous spectrum of the chaotic pulse-position-modulated UWB signal is derived as follows:

(28) $$S^{c}(f)=\frac{1}{T}{\left|W\left(f\right)\right|}^{2}\sum_{l=-\infty }^{+\infty }\left\{E[\beta_{n}(f)\beta_{n+1}^{*}(f)]-E[\beta_{n}(f)]E[\beta_{n+1}(f{)}^{*}]\right\}\exp(-j2\pi flT)\;\begin{array}{cc} = & \frac{1}{T}{\left|W(f)\right|}^{2}\{1-{\left[\frac{\sin(\pi fT_{0})}{\pi fT_{0}}\right]}^{2}\} \end{array}.$$

The discrete spectrum is then expressed as follows:

(29) $$S^{d}\left(f\right)=\frac{1}{T^{2}}\sum_{l=-\infty }^{+\infty }{\left|W\left(\frac{l}{T}\right)\right|}^{2}{\left[\frac{\sin(\pi \frac{l}{T}T_{0})}{\pi \frac{l}{T}T_{0}}\right]}^{2}\delta \left(f-\frac{l}{T}\right).$$

**Figure 9:**
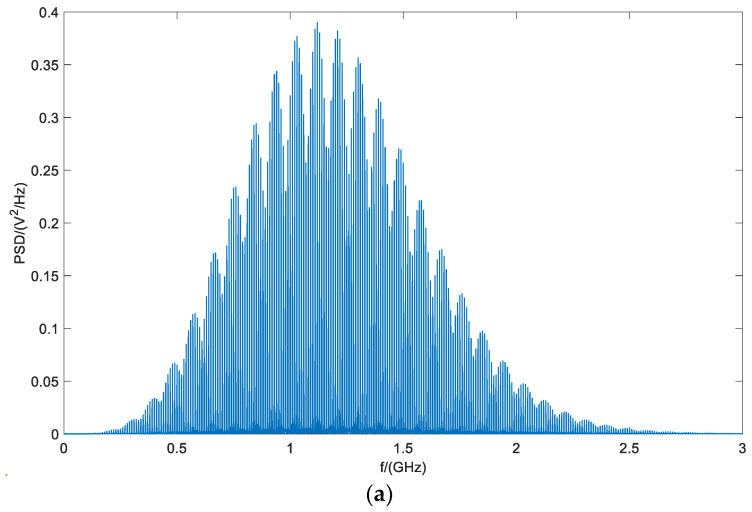

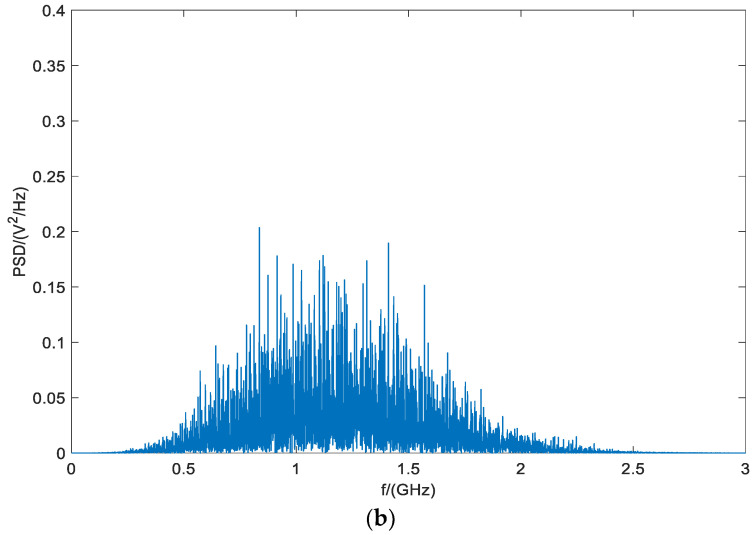
Power spectral density: (**a**) unmodulated; (**b**) chaotic pulse-position modulation.

The introduction of chaotic sequences changes the power distribution of the system; the power that was only represented in the form of discrete spectral lines is partially transferred to the continuous spectrum. The calculated power-spectral densities of the unmodulated and chaotic pulse-position-modulated UWB signals are shown in Figure 9.

Following chaotic modulation, the peak power spectral density (PSD) of the UWB signal decreased by approximately 50%, significantly increasing the difficulty for reconnaissance and interference equipment to detect the fuze signal. Moreover, the modulated UWB fuze signal exhibited a more random frequency distribution. Figure 9a clearly shows a distinct spectral peak, facilitating targeted interference by interference equipment. By contrast, the chaotically modulated UWB signal lacked a prominent spectral peak, making it impractical for interference equipment to target specific frequencies.

#### 3.3.3. Anti-Jamming Performance

In this study, the anti-jamming performance of radio fuzes was evaluated using targeted sinusoidal jamming, which was applied to the fuze according to an established method after accurately obtaining the frequency of the fuze’s signals. The interference signal is expressed as follows:

(30) $$j(t)=a\cos(2\pi f_{J}t+\varphi ),$$

The target echo signal with sinusoidal interference is correlated with the local reference signal in a modulation period, and the output signal of the correlator can be obtained as follows:

(31) $$u_{R}(t)=\int_{T_{r}}[r(t)+j(t)]u(t)dt=\int_{T_{r}}r(t)u(t)dt+\int_{T_{r}}j(t)u(t)dt.$$

The average power of the interference signal at the output of the correlator is as follows:

(32) $$P_{j(t)}=\int_{-\infty }^{\infty }J(f)|H(f){|}^{2}df\;=\int_{-\infty }^{\infty }\left\lceil \frac{a^{2}}{4}\delta (f-f_{J})+\frac{a^{2}}{4}\delta (f+f_{J})\right\rceil |H(f){|}^{2}df\;=\frac{a^{2}}{2}|H\left(f_{J}\right){|}^{2}.$$

According to the literature [37], the gain in signal-to-interference ratio in UWB fuze correlation detection with sinusoidal interference is as follows:

(33) $$G=\frac{SIR_{out}}{SIR_{in}}=\frac{NT_{r}A^{2}E}{{\left|H(f_{J})\right|}^{2}}\approx \frac{NT_{D}A^{2}E}{{\left|H\left(f_{J}\right)\right|}^{2}}\;,$$

where N is the number of pulses; E is the energy of a single pulse; A is the local reference-signal amplitude; $T_{D}$ is the pulse-repetition period; $T_{r}$ is the modulation period, H(f) is the spectrum of the echo signal; and $f_{J}$ is the frequency of the interference signal.

According to Equation (33), when the jamming frequency corresponds to the spectral peak of the delayed signal in the local correlator of the UWB fuze, $H(f_{J})$ reaches its maximum, i.e., the correlation-detection processing gain G is a minimum. At this point, $f_{J}$ is referred to as the optimal jamming frequency.

**Figure 10:**
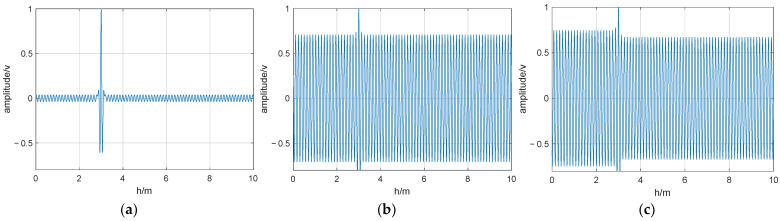
Output waveform of fuze when jamming frequency is optimal: (**a**) chaotic pulse-position modulation; (**b**) M-sequence modulated; (**c**) unmodulated.

**Figure 11:**
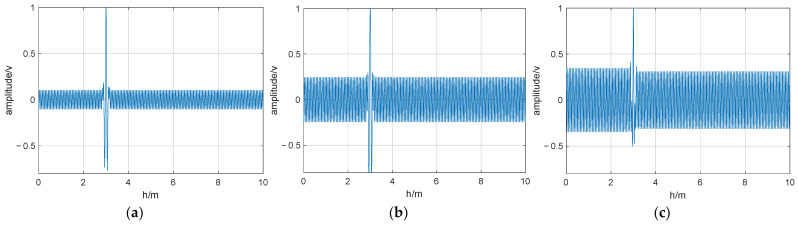
Output waveform of fuze when jamming frequency deviates from optimal value: (**a**) chaotic pulse-position modulation; (**b**) M-sequence modulated; (**c**) unmodulated.

The parameters of the UWB fuze simulation model in this study were as follows: a pulse repetition frequency of 10 MHz; a pulse width of 0.5 ns; the accumulation period of fuze-related processing set to 1 μs; the simulated rendezvous distance of the projectile at 10 m; and a predetermined explosion height of 3 m. Figure 10 and Figure 11 show the correlation-detection output waveforms under optimal and non-optimal interference frequencies.

Without modulation, when the interference-signal frequency was optimal, the interference effect was pronounced, causing the output correlation peaks to be submerged in the interference signal. Away from the optimal frequency, the interference effect decreased significantly, allowing the correlation peaks to be output normally. With M-sequence modulation, the anti-interference effect showed a slight improvement compared to no modulation. However, at the optimal interference frequency, the output correlation peaks were still submerged in the interference signal. By contrast, in the chaotic pulse-position-modulated case, an optimal interference frequency does not exist, and the output correlation peaks can be output normally even when there is an interfering signal present. Thus, the chaotic-pulse-position-modulated UWB fuze demonstrates strong resistance to sinusoidal interference: effective interference is difficult for jammers to achieve, even if they can identify the frequency of the fuze signal.

## 4. Conclusions

This study investigated the RF stealth waveforms of a UWB radio fuze employing chaotic pulse-position modulation. A perturbation signal based on Tent-chaotic mapping was introduced to extend the periods of chaotic sequences without increasing complexity. Through calculations of the approximate entropy and sequence period analysis, and numerical simulations, we confirmed that the performance of the perturbed Tent-chaotic sequence under byte constraints closely matched that of the unperturbed Tent-chaotic sequence under ideal conditions. The chaotic pulse-position-modulated UWB radio fuze significantly outperformed its unmodulated counterpart in terms of detection capability, interception avoidance, and anti-jamming ability. This research offers theoretical insights to enhance the battlefield survivability of radio fuzes through RF stealth technology. The limitation lies in the fact that the study in this paper is based on the comparison of various aspects of the performance of chaotic modulated UWB fuzes with unmodulated UWB fuzes, and in future work, quantitative evaluation will continue to be investigated in order to evaluate the RF stealth performance of radio fuze by means of standardized quantitative criteria.

## Data Availability

The original contributions presented in the study are included in the article, further inquiries can be directed to the corresponding author.
